# A Review of the Theoretical Fascial Models: Biotensegrity, Fascintegrity, and Myofascial Chains

**DOI:** 10.7759/cureus.7092

**Published:** 2020-02-24

**Authors:** Bruno Bordoni, Thomas Myers

**Affiliations:** 1 Physical Medicine and Rehabilitation, Foundation Don Carlo Gnocchi, Milan, ITA; 2 Anatomy, Anatomy Trains International, Walpole, USA

**Keywords:** fascia, myofascial, myofascial chains, fascintegrity, biotensegrety, osteopathic, physiotherapy

## Abstract

The fascial tissue includes solid and liquid fascia (body fluids such as blood and lymph). The fascia's nomenclature is the subject of debate in the academic world, as it is classified starting from different scientific perspectives. This disagreement is not a brake but is, in reality, the real wealth of research, the multidisciplinarity of thought and knowledge that leads to a deeper understanding of the topic. Another topic of discussion is the fascial model to conceptualize the human body, that is, how the fascial tissue fits into the living. Currently, there are some models: biotensegrity, fascintegrity, and myofascial chains. Biotensegrity is a mechanical model, which takes into consideration the solid fascia; fascintegrity considers the solid and the liquid fascia. Myofascial chains converge attention on the movement and transmission of force in the muscle continuum. The article is a reflection on fascial models and how these are theoretical-scientific visions that need to be further investigated.

## Introduction and background

Looking for the first article that names the term fascia in the literature (PubMed), we can find that the first text dates back to 1814 [1]. The article describes how a surgical approach for fracture of the leg (fibula and tibia) resolved pain and inflammation, separating the bands of the muscles and the different tissue layers [1]. The concept behind the article of 1814 is that the fascia is connective tissues, which separates and supports the muscles and movements. Nowadays, little has changed. We find the same assumptions in some statements of international committees and federations. The Federative International Program on Anatomical Terminologies (FIPAT) (2011) defines the fascia: “a sheath, a sheet, or any other dissectible aggregations of connective tissue that forms beneath the skin to attach, enclose, and separates muscles and other internal organs” [2]. Another authoritative organization, the Fascia Nomenclature Committee (2014), defines the fascia: “The fascial system includes adipose tissue, adventitia, neurovascular sheaths, aponeuroses, deep and superficial fasciae, dermis, epineurium, joint capsules, ligaments, membranes, meninges, myofascial expansions, periosteum, retinacula, septa, tendons (including endotendon/peritendon/epitendon/paratendon), visceral fasciae, and all the intramuscular and intermuscular connective tissues, including endomysium, perimysium, epimysium” [3]. Since connective tissue falls within the meaning of fascia, the Foundation of Osteopathic Research and Clinical Endorsement (FORCE), founded in 2013, has added blood and lymph or specialized connective tissue to the fascial nomenclature [4,5]. This group has taken another step forward. The previous definitions have considered the fascia as deriving from the mesoderm embryological tissue (such as muscle tissue). In reality, the fascial tissue of the skull and part of the neck have a double origin, such as the mesoderm and the ectoderm, merging into a continuum [6]. The latest definition of fascia is (2019): “The fascia is any tissue that contains features capable of responding to mechanical stimuli. The fascial continuum is the result of the evolution of the perfect synergy among different tissues, liquids and solids, capable of supporting, dividing, penetrating, feeding and connecting all the regions of the body, from the epidermis to the bone, involving all its functions and organic structures. This continuum constantly transmits and receives mechanometabolic information that can influence the shape and function of the entire body. These afferent/efferent impulses come from the fascia and the tissues that are not considered as part of the fascia in a biunivocal mode. In this definition, these tissues include: "epidermis, dermis, fat, blood, lymph, blood, and lymphatic vessels, tissue covering the nervous filaments (endoneurium, perineurium, epineurium), voluntary striated muscle fibers and the tissue covering and permeating it (epimysium, perimysium, endomysium), ligaments, tendons, aponeurosis, cartilage, bones, meninges, and tongue” [3].

## Review

Fascial models: biotensegrity & fascintegrity

**Figure 1:**
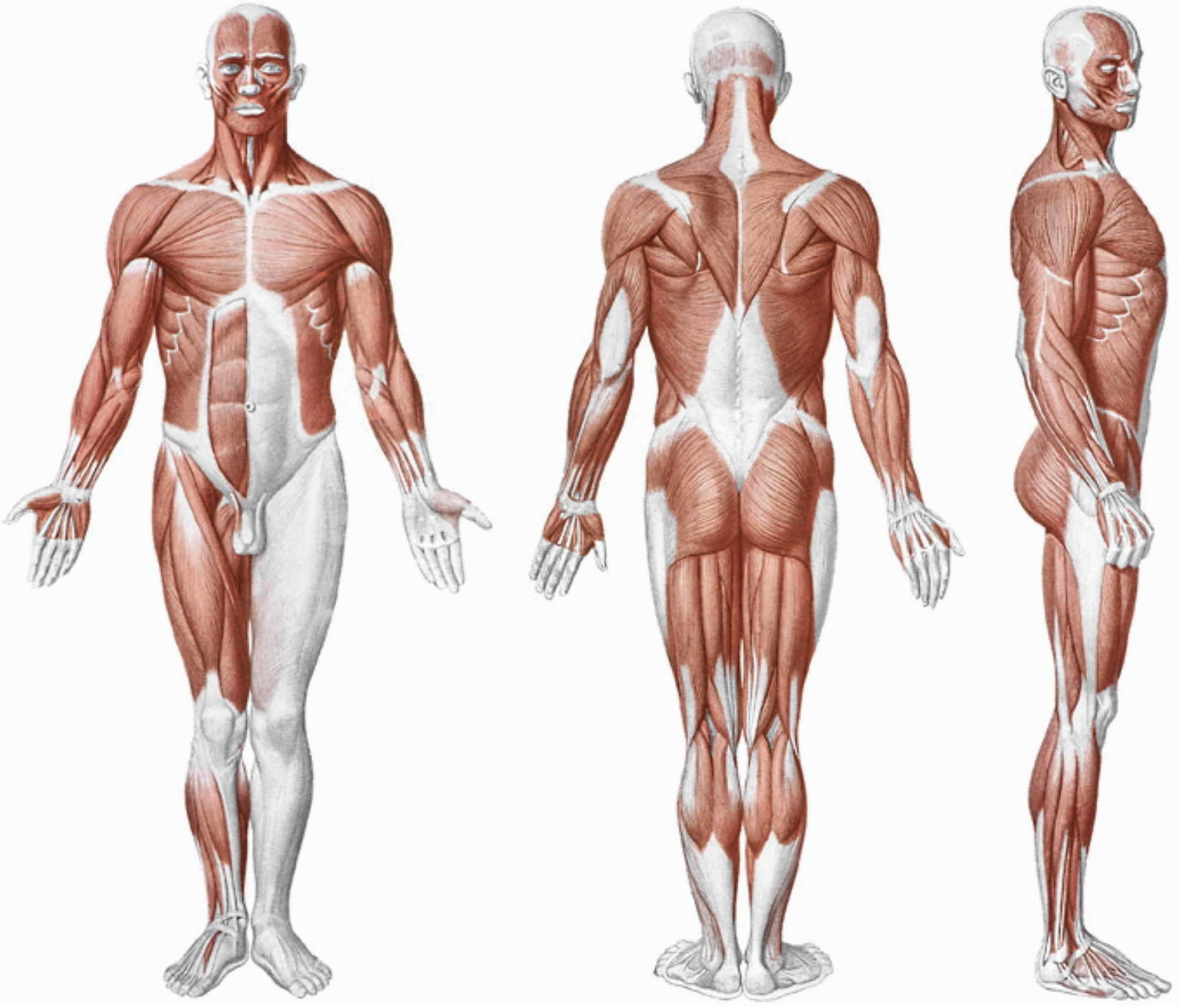
Shape and arrangement of the muscles on the ventral surface (a), dorsal (b) and lateral (c) of the human body Reproduced with permission Anastasi *et a*l. AA VV, Anatomia dell’uomo, fourth edition, 2010, pp76. Editor: Edi-Ermes, Milano [Human Anatomy].

**Figure 2:**
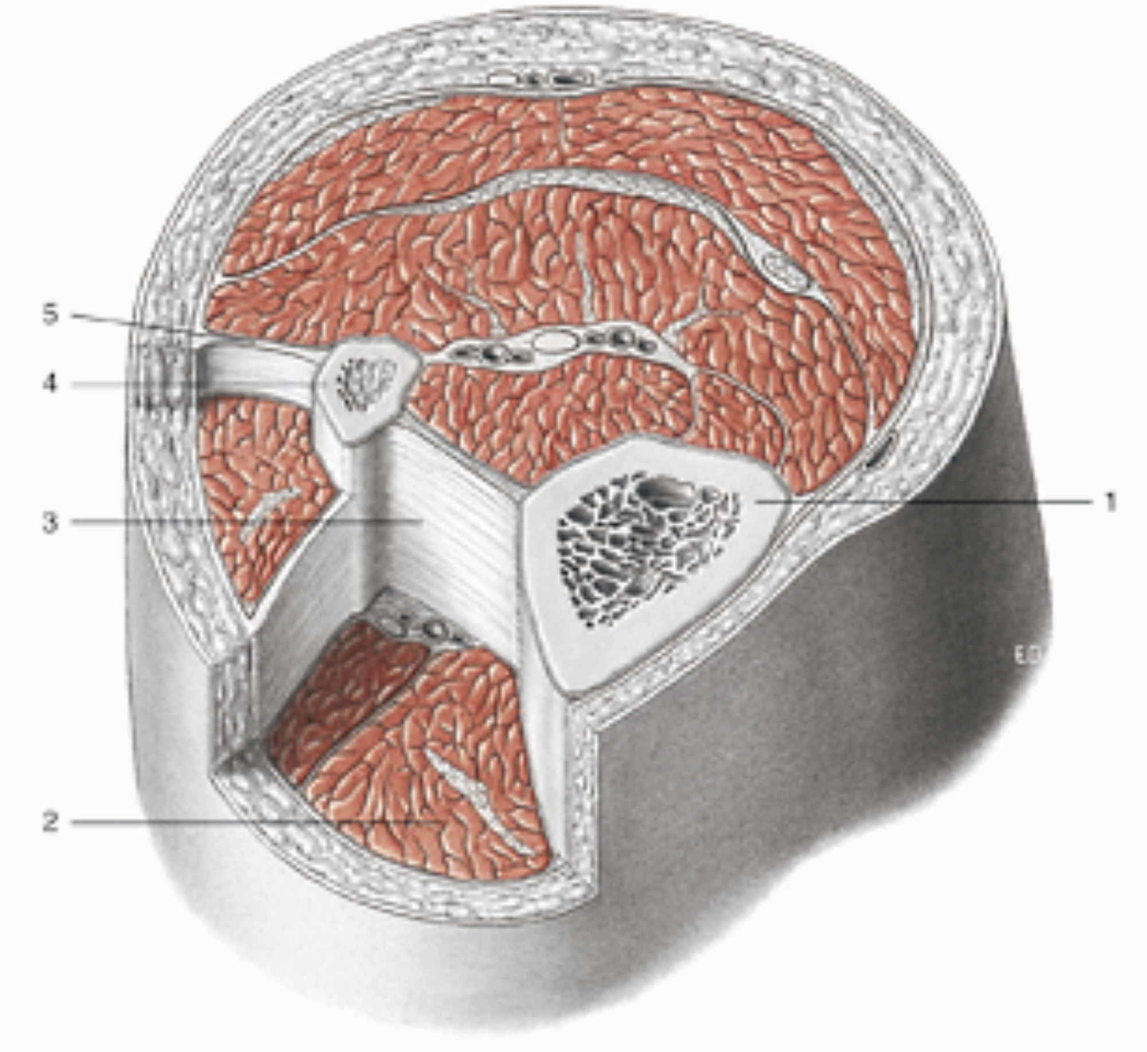
Transverse section at the level of the upper third of the leg Notes: 1 tibia; 2 muscular loggia; 3 interosseous membrane; 4 fibula; 5 intermuscular septum. All tissues are enveloped by fascial continuum. Reproduced with permission Anastasi et al. AA VV, Anatomia dell’uomo, fourth edition, 2010, pp. 89. Editor: Edi-Ermes, Milano [Human Anatomy].

The movement of the human body implies the use of fascial continuum. The human body is based and managed by sensations: emotions, pain, and movement [7-9]. To give an example, the cerebellum considered classically as an area for sorting and managing information used for movement is an important crossroads of information related to pain and emotions [10]. Cerebellar afferents come to affect the corticolimbic networks; the cerebellum is involved in all perceptual information (proprioceptors, nociceptors, interceptors, exteroceptors) as perceptual processing [10]. Chronic altered information from conditions such as pain, depression, lack of movement will negatively affect the cognitive aspect (memory, problem-solving, elaboration of ideas) [11-12]. The fascial continuum allows movement and is a source of information in a reciprocal interrelation, which is capable of influencing also the cognitive aspect [13]. From these concepts, the need arises to frame the fascial system in a model that can represent the living and understand, prevent and possibly cure the dysfunctions that can result from the fascia. To give some examples, there is evidence that eccentric post-contraction muscle pain is mainly attributable to the muscular fascial system; a dysfunctional fascial system could be one of the causes that determine the symptoms of fibromyalgia [14-15]. The discovery of the contraction of fibroblasts and myofibroblasts (1968, 1972) has directed the attention of research towards the fascial tissue, to explode with a large number of publications from the end of the twentieth century [16-17]. Currently, in the scientific panorama, we can find some fascial models: biotensegretive model, fascintegretive model, and myofascial chains. The first model was inspired by a Fuller architectural idea, which first coined the term tensegrity, that is, a structure in tensional equilibrium (constant mechanical tension and non-constant compression) [18]. The next step by Dr Levin was to coin the term biotensegrity, reporting the principle underlying Fuller in the living [19]. The transmission of mechanical tension (active or passive) determines a constant adaptation of the body structure, without damaging or deforming the integrity of the form and function; this concept can be applied to the whole body, to a contractile district and to the single cell (Figures 1, 2) [18].

**Figure 3:**
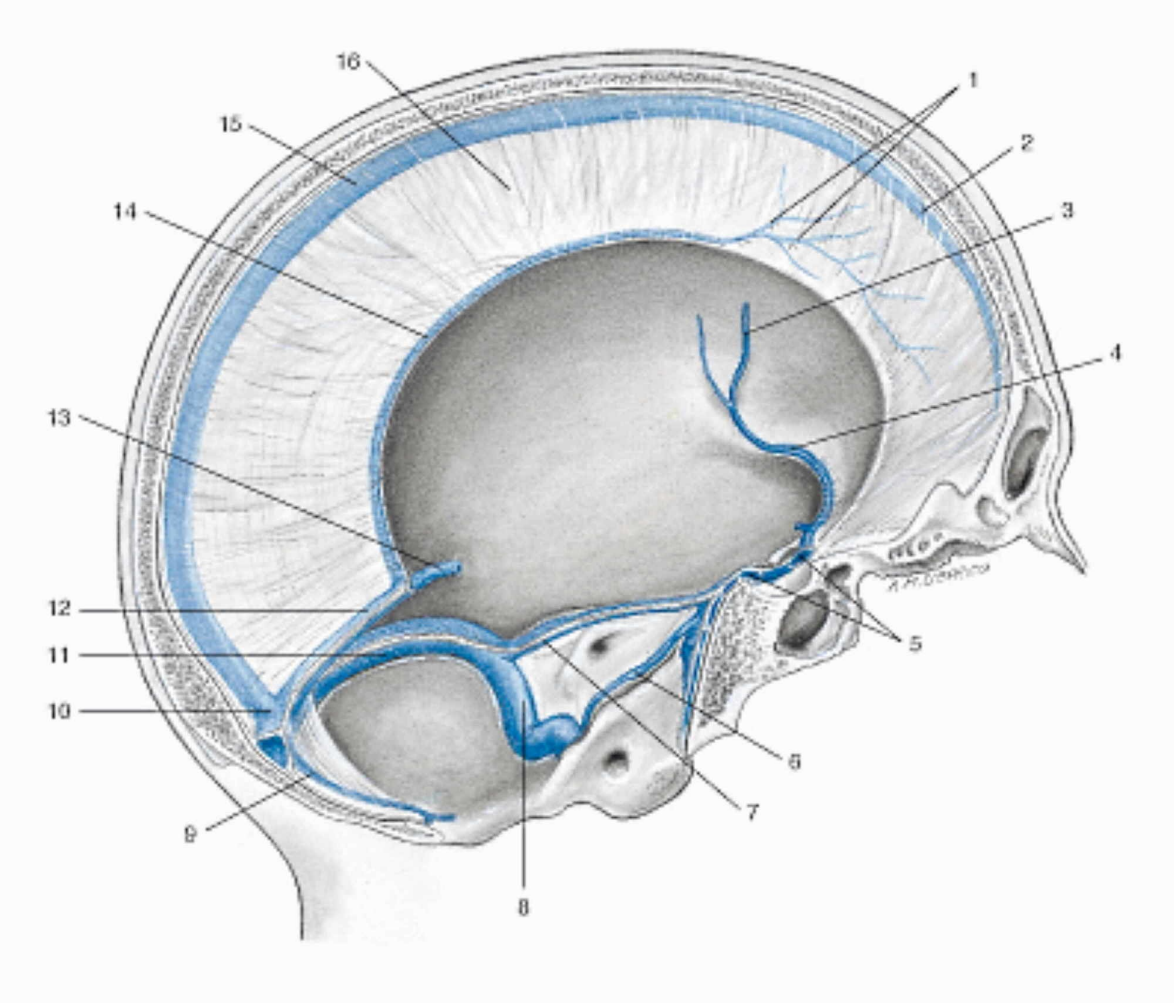
Representation of the venous sinuses of the dura mater in a sagittal section of the skull (liquid fascia) 1 Vein of the dura mater; 7 Left superior petrosal sinus; 12 Straight sinus; 2 Superior sagittal sinus; 8 Left sigmoid sinus; 13 Great cerebral vein (of Galen); 3 Left middle cerebral vein; 9 Occipital sinus; 14 Inferior sagittal sinus; 4 Left sinus sphenoparietal; 10 Confluence of sinuses; 15 Superior sagittal sinus; 5 Intercavernous sinus; 11 Left transverse sinus; 16 Falx cerebri; 6 Left inferior petrosal sinus. Reproduced with permission Anastasi *et al*. AA VV, Anatomia dell’uomo, fourth edition, 2010, pp 432.  Editor: Edi-Ermes, Milano [Human Anatomy].

What is missing from this theoretical mechanical model is the integration of the tension caused by the nervous tissue, the vascular tissue, the movement of the viscera and bodily fluids (liquid fascia), such as blood, lymph, interstitial and intracellular fluids. Body fluids not only allow life and functional continuity but, in particular, they determine the passage of mechanical tensions faster than the muscles and allow the mechanotransductive mechanisms to express themselves in a correct environment [18]. Fluids govern form and function [18,20]. The second theoretical model called fascintegrity not only includes the tissues integrated into biotensegrity but adds fluids, making the fascial continuum more mirroring than what are the modern scientific dictates on cellular and systemic behaviour [18]. What is missing from this model is the contextualization of the emotional sphere and the sphere of pain, as these aspects can influence the human body and the fascial system [21]. Myers elegantly stressed that we need further studies to find a more suitable model for the living, with further research efforts, and that these representations of the fascial continuum (biotensegrity and fascintegrity) are still only models (Figure 3) [20].

Fascial models: myofascial chains

**Figure 4:**
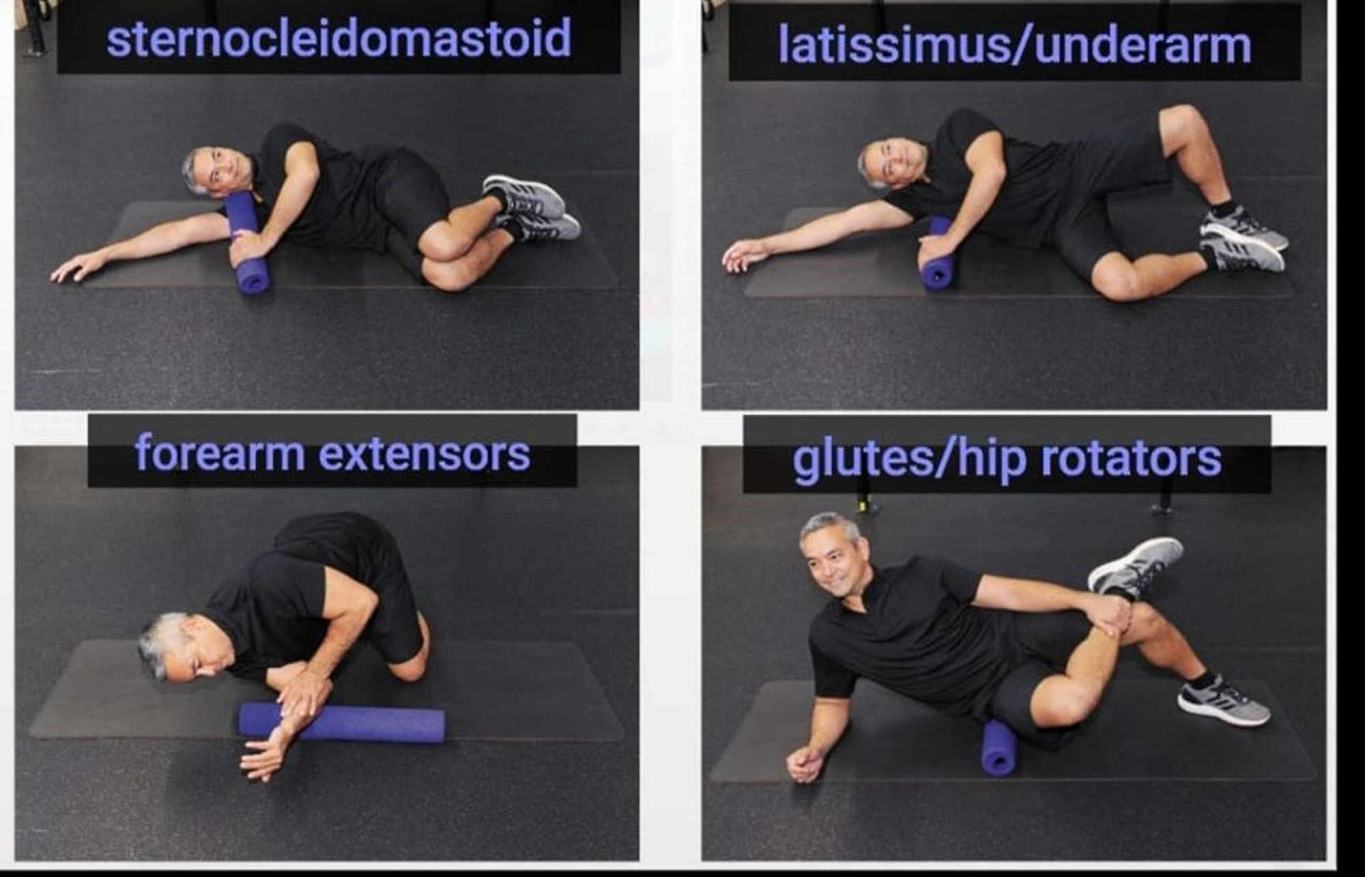
The figure shows the stretching of some parts of the body (the man in the picture is Dr Anthony Chrisco), and elongation is possible only if the whole body adapts to the district to be stretched (with the Fascianator method) Photo was given by Dr Anthony Chrisco.

Myofascial chains perfectly reflect the concept of body continuity. We can find the term muscle chains (chaînes musculaires) by Busquet (1992) and since 1993 with Souchard, arriving in Paoletti with the fascial chains (Les Fascias) in 1998 [22-23]. Myers laid the foundations for myofascial chains in 1997, while Stecco in 1988 saw muscle continuity and acupuncture meridians [22-23]. The concept of myofascial chains, that is, the tension of a contractile district has repercussions and influences other districts near and far, is used in different disciplines, from physiotherapy to yoga, from sport to meditation (Figure 4) [24-28].

**Figure 5:**
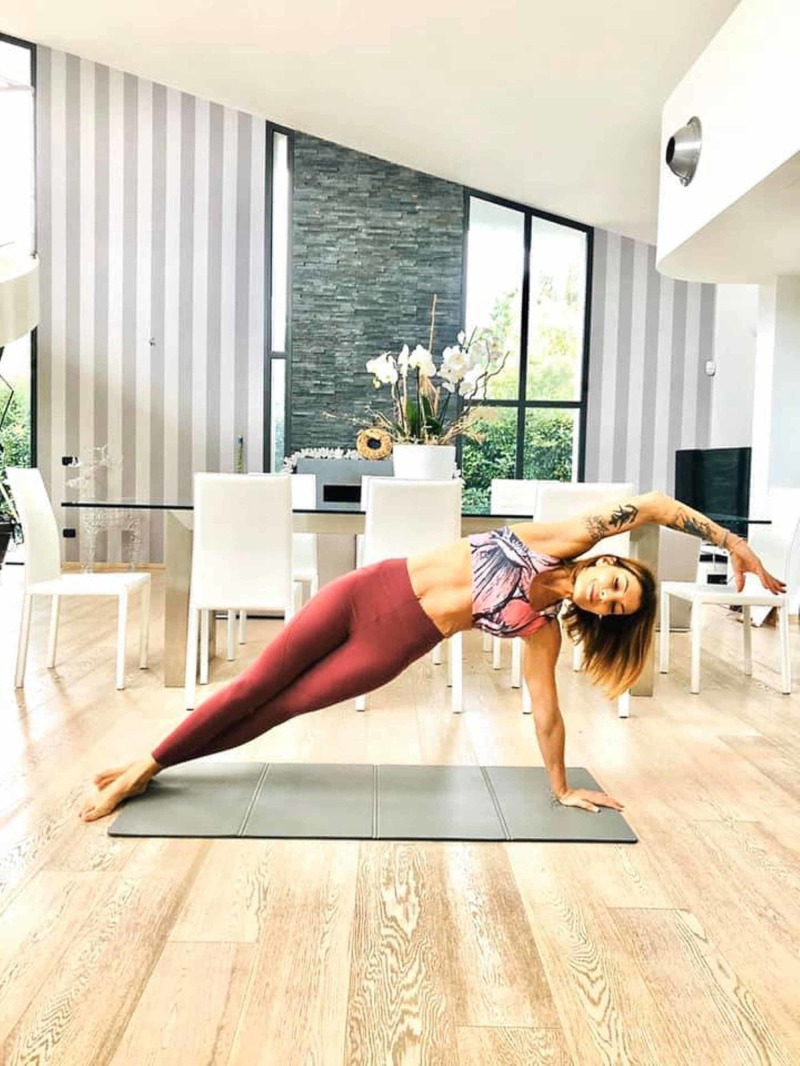
The figure shows how the whole body is held in tension to maintain the position, demonstrating the myofascial continuum (the woman in the picture is Dr Francesca Skyola) The photo was given by Dr Francesca Skyola.

From a microscopic and macroscopic point of view, it has been shown that myofascial tissue (muscle and connective tissue) can transmit the tension produced to other muscles, through animal, cadaver and in vivo studies [23,29-31]. At a microscopic level, the muscle cell can implement different strategies, based on the morphology of the muscle. In spindle-shaped muscles, the sarcomere that contracts or stretches, transmits the tension to the costamers and to the sarcomeres in series, which bring the tension to the sarcolemma; sarcolemma transmits force through transmembrane proteins (group of integrins and others) towards the extracellular matrix and, finally, towards the endomysium and the tendon [32-34]. The transmission speed from sarcomeres to the tendon is fast but dispersive; the biceps muscle is faster but less strong than the deltoid muscle [35]. If the muscle is pennate or multi-pennate, the distribution of force is different. The production of tension by sarcomeres travels mainly from sarcomere to parallel sarcomere, as the fibers are not in line with the tendon but are oblique with respect to the longitudinal axis; the speed is slower but, in this mode, the force expressed is greater [35-36]. In the fusiform muscle, the distribution is longitudinal, while in the pennate muscle the distribution is first parallel and then longitudinal [35-36]. The tension produced is carried to other neighbouring muscle districts; this mechanism is called intermuscular myofascial force transmission [33-34]. The tension produced by muscle towards non-muscular structures is called extramuscular myofascial force transmission [37]. The distribution of myofascial tension between the agonist and antagonist muscles takes the two mechanisms simultaneously in perfect balance (Figure 5) [38].

**Figure 6:**
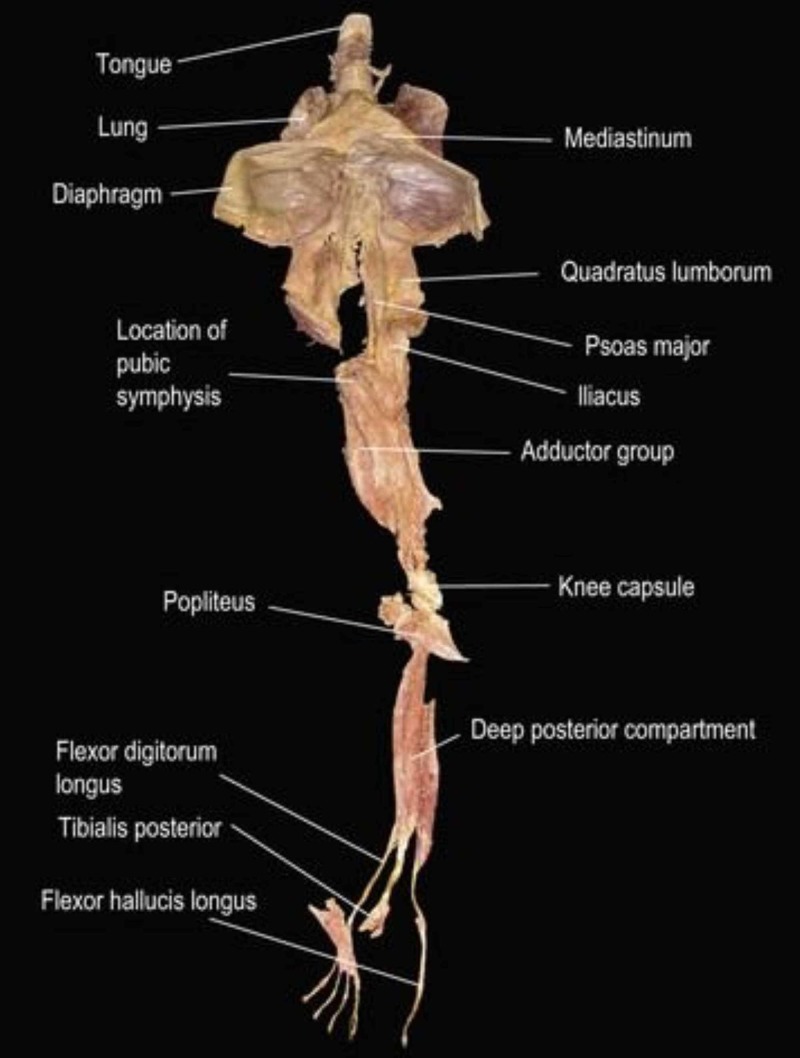
The figure illustrates the deep front line, which shows the continuous connection of myofascial tissue, from the muscles of the toes to the tongue The image is taken from Thomas Myers' book: Anatomy Trains. Myofascial Meridians for Manual & Movement Therapists. Editor: Churchill Livingstone, 2013. Courtesy of Thomas Myers, co-author of the article.

From a macroscopic point of view, revision and cadaver studies (with mechanical or manual traction) have highlighted and demonstrated the existence of some myofascial lines [39-41]. To give an example, stretching, with healthy people, of the lower limbs improves the range of motion of the cervical tract in flexion/extension (Figure 6) [42-43].

There is not always agreement on the existence of myofascial chains in the different planes of the body [44]. The reasons are manifold. The lack of in-depth studies on all body fascial chains, the difficulty of studying these myofascial connections in vivo, and the difficulty in really understanding how these chains combine in cadaver studies, as, very often, the processing for examining the cadaver involves the loss of the maximum movement capacity of the tissues and electrical activity [39-40]. We do not know the neurological connections between the antagonist and agonist muscles of the whole body continuum [45]. We still have to consider factors that can influence the outcome of a search. Hydration and fluids strongly influence the tension produced by the myofascial tissue, even before the cell contraction of fibroblasts and fibroblasts; mechanical stiffness depends primarily on water and fluids [20,46]. The connective tissue can vary its tension within minutes, and this event could alter the results of the research, as well as the different percentage of muscle fibers and the different angle of the fibers with respect to the longitudinal axis of the muscular district, in relation age, sporting activity or the presence of diseases [47]. The research does not consider in depth the presence of fascial transversal connections to the muscular districts, such as the lacertus fibrosus between the biceps brachial and the antebrachial fascia or other fascial structures not necessarily in a "logical" position with respect to the lines of force of the muscles, such as the fascia of Osborne, the arcade of Struthers [48-49]. Subjective anatomical variations must be considered, which can influence intermuscular transmission, as well as the relationship of the viscera with the muscles by means of connective connections. For example, the diaphragm muscle is closely related to the liver via the Glisson capsule, related to the duodenum, stomach, and esophagus [50]. We should take into consideration the fact that the chest musculature is in close contact with the peri-bone fascia (in front of the ribs/sternum and behind the ribs/sternum) or, the fact that the movement of the diaphragm affects the anterior and posterior fascial system of the trunk [50]. We do not know how alteration of the chest or diaphragm in the presence of systemic pathologies (pulmonary, cardiac) is able to alter the relationships between local and distant contractile districts. Emotions can alter the responses of the fascial continuum and we still have no elements in hand to understand how an emotional status can influence the relationship of the myofascial chains of the body [13].

## Conclusions

The models that try to represent the human body through the fascial continuum (biotensegrity, fascintegrity, and myofascial chains) are based on valid concepts but which, currently, do not include all the nuances of the living. For this reason, we must remember that they are theoretical representations that need further steps forward to better understand the fascia and to understand the fascial behavior in the presence of subjective anatomical anomalies and in the presence of pathologies, local and systemic. We need to know how the histology of the fascia changes based on age, based on the daily intake of drugs and how these variables can affect the adaptation of the myofascial continuum.
